# Toward Suppressing Oil Backflow Based on a Combined Driving Waveform for Electrowetting Displays

**DOI:** 10.3390/mi13060948

**Published:** 2022-06-15

**Authors:** Zhengxing Long, Zichuan Yi, Hu Zhang, Jinpu Lv, Liming Liu, Feng Chi, Lingling Shui, Chongfu Zhang

**Affiliations:** 1 College of Electron and Information, University of Electronic Science and Technology of China, Zhongshan Institute, Zhongshan 528402, China; mikaellzx@163.com (Z.L.); 202021020727@std.uestc.edu.cn (H.Z.); 15361320481@163.com (J.L.); liulmxps@126.com (L.L.); chifeng@semi.ac.cn (F.C.); cfzhang@uestc.edu.cn (C.Z.); 2 School of Information and Optoelectronic Science and Engineering, South China Normal University, Guangzhou 510006, China; shuill@m.scnu.edu.cn

**Keywords:** electrowetting display, charges trapping effect, aperture ratio, driving waveform, oil backflow

## Abstract

Electrowetting display (EWD) is a new type of paper-like reflective display based on colored oil, which has gradually become one of the most potential electronic papers with low power consumption, fast response, and full color. However, oil backflow can occur in EWDs, which makes it difficult to maintain a stable aperture ratio. In order to improve the stability of the aperture ratio of EWDs, a new driving waveform was proposed based on analyzing the phenomenon of oil backflow. The driving waveform was composed of a shrinking stage and a driving stage. Firstly, a threshold voltage of oil splitting was calculated by analyzing the luminance curve of EWDs, which were driven by different direct current (DC) voltages. Then, an exponential function waveform, which increased from the threshold voltage, was applied to suppress oil splitting. Finally, a periodic signal combined with a reset signal with a DC signal was applied during the driving stage to maintain a stable aperture ratio display. Experimental results showed that the charge trapping effect could be effectively prevented by the proposed driving waveform. Compared with an exponential function waveform, the average luminance value was increased by 28.29%, and the grayscale stability was increased by 13.76%. Compared to a linear function waveform, the aperture ratio was increased by 10.44% and the response time was reduced by 20.27%.

## 1. Introduction

The concept of electrowetting displays was first proposed by G. Beni in 1983 [1,2]. In 2003, an electrowetting display (EWD) based on the principle of electrowetting was proposed by Hayes [3]. Different driving voltages were used to control the movement of oil, which could achieve an optical display effect of color switching between oil and substrate [4]. As a new type of reflective display, EWDs effectively compensated for the shortcomings of traditional electronic papers in video playback and full-color display [5,6,7,8,9]. However, a low aperture ratio could be caused by oil backflow [10]. Therefore, suppressing oil backflow is an important research topic for EWDs.

Electrowetting is a phenomenon in which the wettability of solid–liquid is changed by applying a voltage between substrates, thereby deforming and displacing droplets [11]. The aperture ratio of EWDs reflects the degree of oil shrinking under the action of the applied voltage, which is depending on fluid materials and pixel structures [12,13,14,15]. The driving waveform consists of a driving voltage sequence, which plays a key role in oil shrinking for a stable gray scale display. However, due to the presence of oil splitting, oil oscillation, and charges trapping in EWDs, they lead to a reduction of aperture ratio and display stability [16,17,18]. In studies of suppressing oil splitting, a method that was used to effectively reduce oil splitting by adding a voltage rising gradient was proposed [19]. However, the response time of EWDs was correspondingly increased. In order to solve this problem, a combined pulse driving waveform was proposed [20]. During the rising stage, the voltage was driven from the threshold voltage to a maximum voltage, which effectively shorten the response time. Furthermore, a stable aperture ratio was maintained by suppressing oil splitting. At the same time, a driving waveform with a quadratic function combined with a square wave was proposed, and a quadratic function waveform was used to suppress oil splitting in the rising stage [21]. The square waveform was applied during the driving process to keep the aperture ratio. This method could not only shorten the response time but also improve the oil splitting. In the study of reducing oil oscillation, an amplitude–frequency hybrid modulation method was proposed, in which the oil was driven by a high voltage to reach the crack, and then the voltage was reduced to stabilize the oil [22]. This method improved the response speed of displaying target grayscales while reducing oil oscillation. In addition, a driving waveform combined with a direct current (DC) and an alternating current (AC) was proposed [23]. The characteristic of oil stability was analyzed, and the oil oscillation was greatly reduced by using this method. In studies of suppressing charge trapping, a driving waveform with a reset signal was proposed [24]. The driving waveform was composed of a driving stage and a reset stage. The driving stage was driven by a DC signal, which was used to turn on pixels. The reset stage was driven by square wave signals of different amplitudes. The negative voltage was used to release captured charges, and the positive voltage was used to drive recovery, which could effectively suppress oil backflow. In addition, an error diffusion algorithm based on optimizing driving waveform was proposed for suppressing oil backflow [25]. These excellent research results provided a reference for us to design a new driving waveform of EWDs.

In this paper, a combined driving waveform was proposed to optimize the aperture ratio of EWDs. The driving waveform consisted of a shrinking stage and a driving stage. The shrinking stage was designed by analyzing the principle of EWDs and the Young–Lippmann equation, and the driving stage was designed according to the charge trapping theory.

## 2. Driving Principle of EWDs

### 2.1. Principle of EWDs

**Figure 1:**
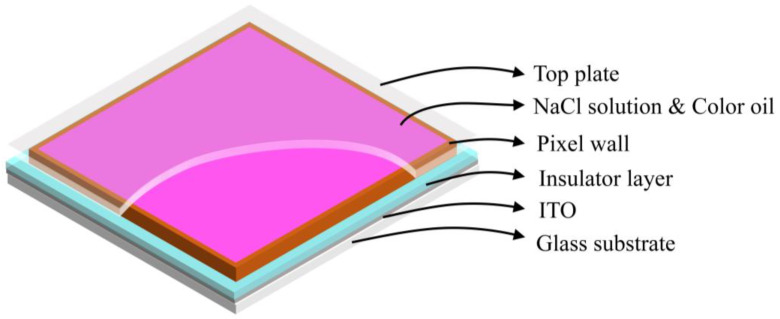
The schematic diagram of a pixel structure in EWDs. It is composed of a top plate, polar liquid (NaCl solution), color oil, pixel walls, an insulating layer, ITO electrodes, and a glass substrate.

Pixels in an EWD are mainly consisted of indium tin oxide (ITO) electrodes, a glass substrate, a top plate, pixel walls, color oil, polar liquid (NaCl solution), and an insulating layer [26,27,28], as shown in Figure 1. When no driving voltage is applied on ITO electrodes, the oil in a pixel is laid between the polar liquid and the hydrophobic insulation layer. Therefore, the pixel displays the color of oil, and the EWD is in an “off” state. When an external voltage is applied to ITO electrodes, the equilibrium state in pixels is broken and the wettability on the surface of the insulation layer is changed. The polar liquid contacts with the insulating layer and oil are pushed to the corners of pixels by the polar liquid and the color of the substrate is shown. At this point, the EWD is in an “on” state. Therefore, the grayscale display can be controlled by adjusting the driving voltage on ITO electrodes. In addition, the aperture ratio is defined as the area ratio between the glass substrate exposed after oil shrinking and the whole pixel grid, which is one of the important parameters for characterizing the performance of EWDs, the expression is shown in Equation (1) [4].

(1) $$W_{AR}\left(V\right)=\left[1-\left(\frac{S_{oil}\left(V\right)}{S_{pix}}\right)\right]\times 100\%$$

where $W_{AR}$ is the aperture ratio of EWDs, and $S_{oil}$ is the area of oil shrinking in a pixel. $S_{pix}$ is the area of a whole pixel, and V represents the driving voltage that is applied on ITO electrodes. The area of pixel walls can be ignored in calculating the aperture ratio. In addition, the movement of oil is mainly affected by interfacial tension and electrostatic force. The contact angle of oil follows the Lippmann–Young equation, and the contact angle can be controlled by adjusting the driving voltage, as shown in Equation (2) [23].

(2) $$\cos\theta =\cos\theta_{0}+\frac{\varepsilon_{0}\varepsilon_{EP}}{2d\gamma_{OW}}V^{2}$$

(3) $$V_{bd}=\sqrt{\frac{2\left(1-\cos\theta \right)\gamma_{OW}}{\frac{1}{4\pi k}\frac{\varepsilon_{oil}S_{oil}}{h}+\frac{\varepsilon_{EP}S_{pix}}{d}}}$$

where θ represents a Lipmann contact angle, and $\theta_{0}$ indicates an equilibrium contact angle between oil and the insulation layer. $\varepsilon_{0}$ is a dielectric constant in a vacuum, $\varepsilon_{EP}$ is a dielectric constant in an insulating layer, and $\varepsilon_{oil}$ is the dielectric constant of oil. k is the electrostatic constant, d indicates the thickness of the insulation layer, and h is the thickness of the oil. $\gamma_{OW}$ indicates oil-liquid interfacial tension, and V is the driving voltage applied on ITO electrodes. $V_{bd}$ is the breakdown threshold voltage of the insulating layer. It can be seen from Equation (3) [29] that $V_{bd}$ is proportional to the thickness of the insulating layer. The maximum driving voltage of the driving waveform cannot be greater than $V_{bd}$.

### 2.2. Charges Trapping of EWDs

**Figure 2:**
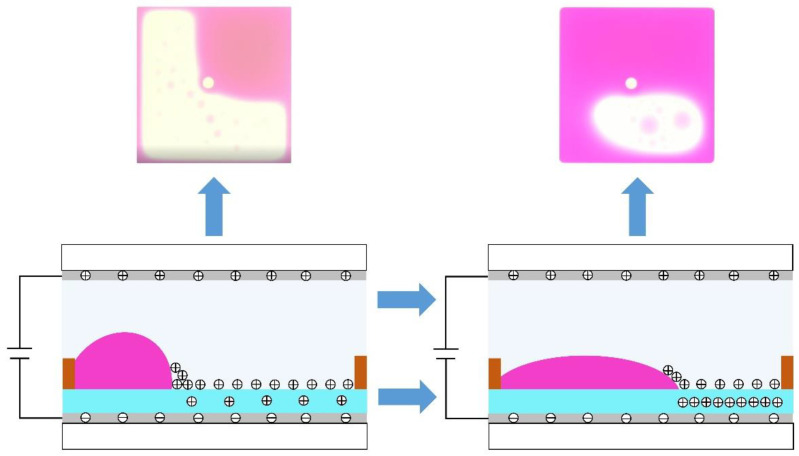
The situation of oil backflow when a driving voltage is applied to a pixel. Due to continuous driving voltages, the number of charges accumulated by the insulation layer also increases correspondingly, resulting in a reduction of the electric field force, and the contracted oil cannot be maintained in a stable state. Therefore, the oil will be gradually spread out on the insulation layer.

According to the Lippmann–Young equation, the contact angle of oil is positively correlated with the square of driving voltages applied to a pixel. However, the trapped charges in the insulation layer cannot be fully released. This will result in a slow response speed and an unstable grayscale display [30,31]. The reason is that part of the charge enters the insulation layer and is trapped when the external voltage is applied. The charges are concentrated at a three-phase contact line of oil, and the local electric field is distorted, resulting in an inability to maintain a balance between Maxwell pressure and Laplace pressure. Therefore, the Lippmann–Young equation needs to be modified. The modified Lippmann–Young equation can be expressed as Equation (4) [29].

(4) $$\cos\theta =\cos\theta_{0}+\frac{\varepsilon_{0}\varepsilon_{EP}}{2d\gamma_{OW}}{\left(V-V_{T}\right)}^{2}$$

where $V_{T}$ is a potential generated by the charges trapping effect. Due to charge trapping, the electrostatic force is reduced for oil. At the same time, with a constant voltage, the hydrophobicity of the insulation layer is enhanced, resulting in oil backflow, which reduces the aperture ratio of EWDs. The oil backflow phenomenon is shown in Figure 2. The contracted oil cannot be maintained in a stable state, and oil will be gradually spread out on the insulation layer. In addition, by analyzing Equation (4), it can be seen that charge trapping is related to the driving voltage. Therefore, the effect of the driving voltage on charge trapping should be taken into account in the design of driving waveforms.

## 3. Experimental Results and Discussion

### 3.1. Experimental Platform

**Figure 3:**
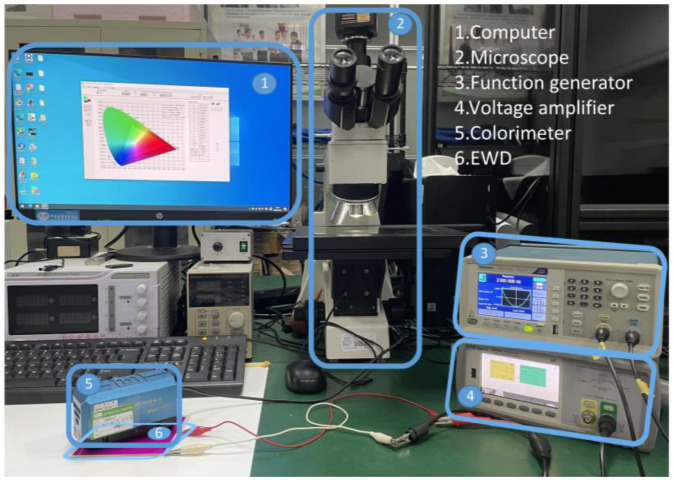
The experimental platform for testing the performance of EWDs. It was composed of a computer, a microscope, a function generator, a voltage amplifier, a colorimeter, and an EWD. The EWD was used as a testing object. The computer, the function generation, and the voltage amplifier were used to generate driving waveforms. Then, the colorimeter was used to obtain the luminance value of the EWD, and the microscope was used to obtain the aperture ratio of the EWD.

In order to measure the parameters of the driving waveform and evaluate its performance, a complete optical experimental platform was built to record the display status of pixels and measure the luminance value of EWDs. As shown in Figure 3, the experimental platform mainly includes: (1) a computer (Pro G6, HP, Beijing, China); (2) a microscope (SZ680, Chongqing Optec Instrument Co., Ltd., Chongqing, China), which was used to measure aperture ratio of EWDs; (3) a function generator (AFG1022, Tektronix, Beaverton, OR, USA), which was used to generate driving waveforms. (4) a voltage amplifier (Agilent 33502A, Agilent, CA, USA); (5) a colorimeter (Arges-45, Admesy, Ittervoort, The Netherlands), which was used to record the luminance value of EWDs. 

The EWD panel size was $10\times 10\;{\mathrm{cm}}^{2}$, and the color of the oil was magenta. The resolution of the EWD was 320×240. The size and height of pixels were $150\times 150\;{\mu m}^{2}$ and 18 μm, respectively. The thickness of the ITO layer and the insulator layer was 2.5 nm and 1 μm, respectively.

The detailed experimental process was as follows. First, waveform files were edited by the computer and imported into the function generator by serial communication. Second, the required driving waveform was generated, which was amplified by the amplifier to drive the EWD. Then, the colorimeter was used to measure the luminance value of the EWD in real time. Finally, the microscope was used to record pixel images and the software was used to calculate the aperture ratio.

### 3.2. Proposed Driving Waveforms

**Figure 4:**
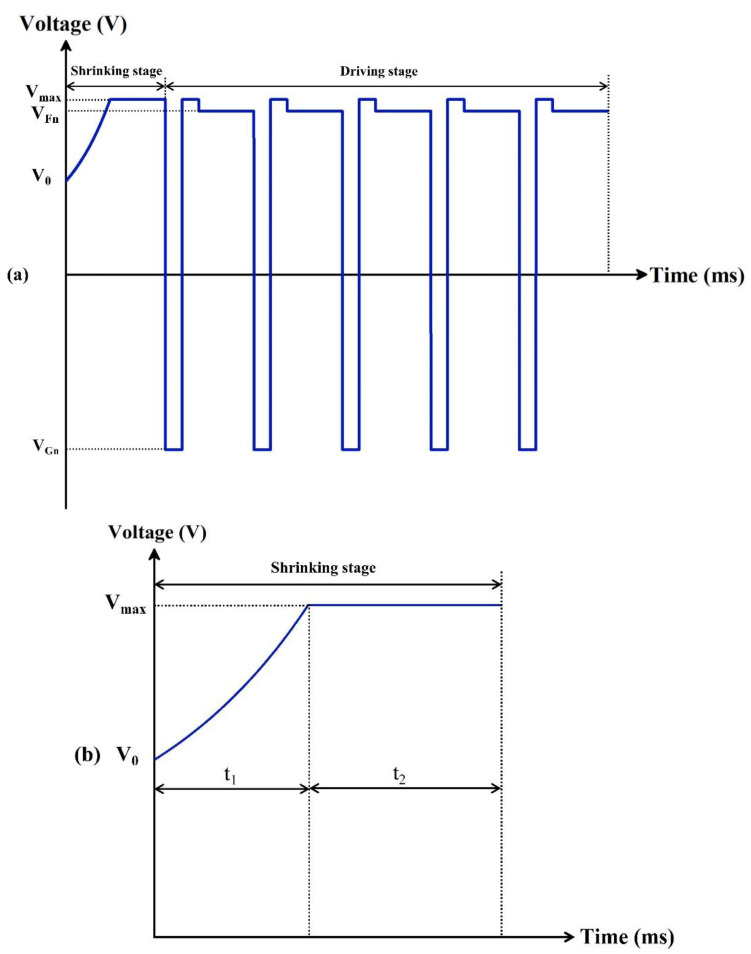

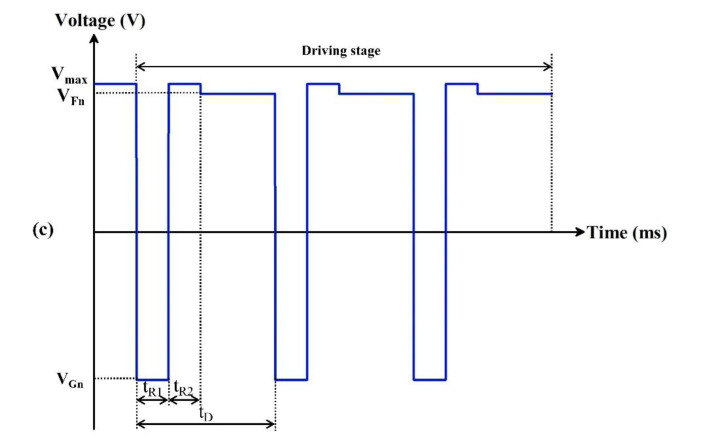
Driving scheme for suppressing oil backflow. (**a**) A complete schematic diagram of the proposed driving waveform. It was composed of a shrinking stage and a driving stage. $V_{Gn}$ was a release voltage in the reset signal. $V_{0}$ was a threshold voltage of the shrinking stage. $V_{Fn}$ was a DC voltage of the target grayscale. $V_{max}$ was a voltage at which oil completely shrunk. (**b**) An example of driving schemes that could be used to prevent oil splitting. $t_{1}$ was the driving time of the exponential function waveform. $t_{2}$ was the driving time of the DC voltage. (**c**) An example of driving schemes that could be used to suppress oil backflow. $t_{R1}$ and $t_{R2}$ were the driving time of the positive voltage and the negative voltage of the reset signal in the driving stage, respectively. $t_{D}$ was the period of a reset signal applied in the driving stage.

Since pixels in EWDs were affected by charge trapping, it was difficult to maintain a stable aperture ratio. In order to obtain a higher aperture ratio and stable luminance value, a new driving waveform was proposed, which consisted of an exponential function and an AC reset signal. As shown in Figure 4, it included a shrinking stage and a driving stage.

During the shrinking stage, the oil was maintained a free diffusion equilibrium state when the driving voltage was less than $V_{0}$. Therefore, an initial voltage of the shrinking stage was set to solve this problem. Then, the driving voltage was raised from the initial voltage to a high-level voltage of the shrinking stage in an exponential function waveform. Because the exponential function waveform could effectively prevent oil splitting caused by the voltage mutation, which could improve the aperture ratio of EWDs. The exponential function could be expressed by Equation (5).

(5) $$V=\left(V_{0}-1\right)+e^{\beta t}$$

where V was the real-time driving voltage of the oil shrinking stage, $V_{0}$ was the threshold voltage of oil shrinkage, β was a time constant of the exponential function, and the relationship between the driving voltage and the driving time could be expressed by Equation (6).

(6) $$V^{’}=\beta e^{\beta t}$$

where $V^{’}$ was a rising rate of the driving voltage. It could be seen that β was proportional to the driving voltage increase rate when the driving time was unchanged. Therefore, the rising rate of the driving voltage could be controlled by adjusting β, which could suppress oil splitting and reduce the driving time. 

In the driving stage, a combined waveform was designed. It consisted of a reset signal and a driving signal, the reset signal could be divided into a charge release phase and a driving recovery phase. In the driving recovery phase, a better grayscale was obtained by the maximum driving voltage, which was set to $V_{max}$. $V_{Fn}$ represented the DC voltage that was required to drive pixels to grayscale, it could be determined by measuring the voltage characteristic of EWDs. $V_{Gn}$ represented a negative voltage used in the charge release phase. f represented the driving frequency of the driving stage. $t_{D}$ represented the period where the reset signal was applied. Durations of the negative voltage and the positive voltage in the reset signal were represented by $t_{R1}$ and $t_{R2}$, respectively. $t_{R1}+t_{R2}$ represented the duration of the reset signal in one driving cycle. The reset signal was used to suppress the charge trapping effect for maintaining the driving voltage on ITO electrodes effectively, and the driving signal was used to control the state of pixels. In this way, not only the polarization phenomenon was avoided, but also the equilibrium state of oil could be maintained in the driving stage.

### 3.3. Testing of the Shrinking Stage

**Figure 5:**
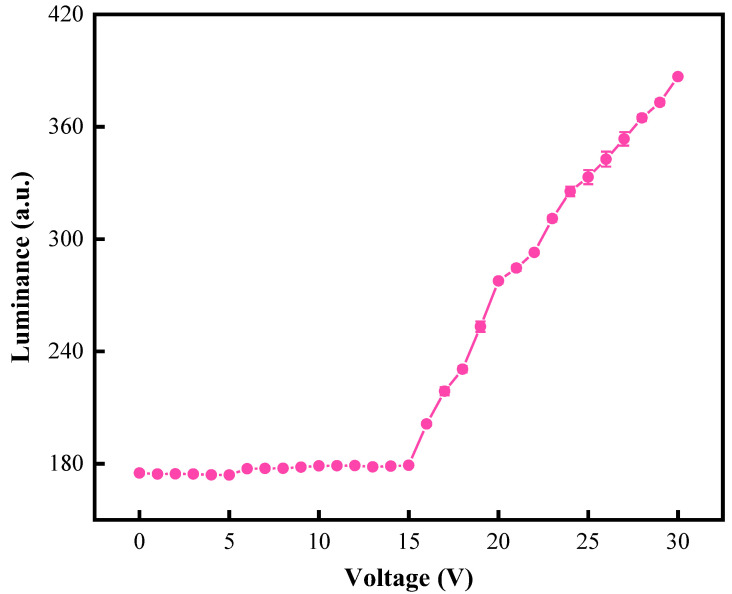
The luminance curve of an EWD when it was driven by different DC voltages. The luminance value was 178.834 when the DC voltage was less than 15 V. The luminance value started to increase when the DC voltage was 16 V. The maximum luminance value was obtained when the DC voltage was 30 V.

In order to determine the threshold voltage of oil splitting, $V_{0}$ and $V_{max}$ were tested by adjusting DC voltages. The driving range of DC voltages was set to 0–30 V, and the change of luminance value is shown in Figure 5. When the voltage was 0–15 V, the luminance value was almost unchanged. The color displayed by pixels was the color of oil. When the driving voltage reached 16 V, oil was beginning to split. The luminance value was raised rapidly, and the EWD changed from the color of oil to the substrate. The luminance was increased with the increase in the driving voltage, and the increase rate gradually tended to be gentle. In order to avoid damage to the insulation layer due to a high driving voltage, $V_{max}$ was set to 30 V and the threshold voltage was set to 16 V.

**Figure 6:**
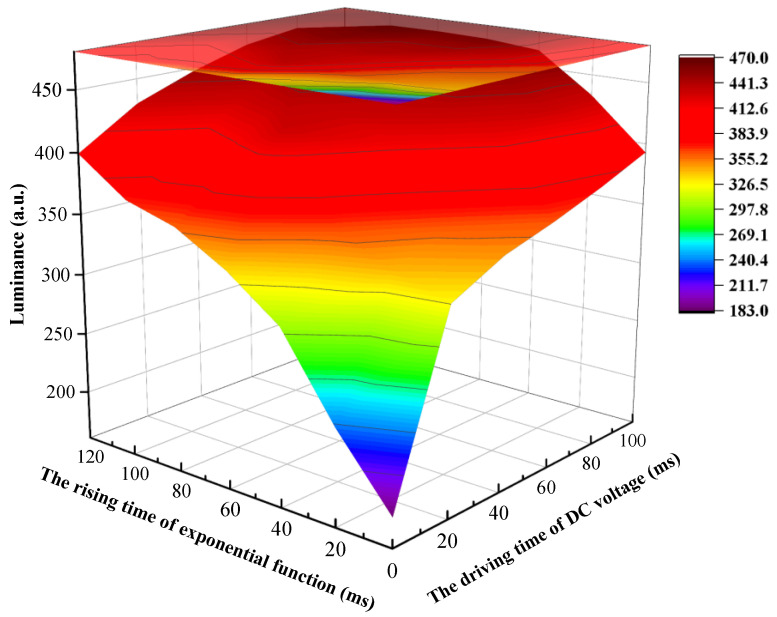
The luminance value was changed with different *t*_1_ and *t*_2_. When *t*_1_ was less than 80 ms, the luminance value was low because of serious oil splitting. The luminance value tended to be saturated as time increased. A saturated luminance value could be obtained when *t*_1_ was set to 80 ms and *t*_2_ was set to 100 ms.

In the shrinking stage, the exponential function voltage was used to prevent oil splitting, DC voltage was used to drive oil to shrink quickly. Then, the influence of exponential function rising time and DC voltage driving time on oil splitting were analyzed, respectively. The rising time $t_{1}$ was set to 0–120 ms and the driving time $t_{2}$ was set to 0–100 ms. The luminance variations between different rising times and driving times is shown in Figure 6. It could be seen that the luminance value gradually increased with the increase in $t_{1}$ and $t_{2}$, indicating that the degree of oil split decreased during the driving process, and the degree of shrinking increased steadily. When $t_{1}$ was less than 80 ms, due to a high $V^{\prime }$, the internal electric field intensity applied to EWDs increased rapidly, and oil was severely split. As the driving time increased, the luminance value could not reach saturation. When $t_{1}$ was set to 80–120 ms, the oil splitting phenomenon could be improved, but a long $t_{2}$ was still needed to recombine the oil. When $t_{1}$ was greater than 80 ms, as $t_{2}$ increased, the luminance value also gradually increased and tended to saturate. It could be proven that oil could be effectively prevented from splitting by applying an electric field with a long driving time. When $t_{1}$ was 80 ms and $t_{2}$ was 100 ms, the luminance value tended to saturate, and the maximum value was 469.577. Therefore, $t_{1}$ was set to 80 ms and $t_{2}$ was set to 100 ms.

### 3.4. Testing of the Driving Stage

Due to the charges trapping effect, charges stored in the insulating layer could not be released, which resulted in oil backflow, and the aperture ratio was decreased. Therefore, a reset signal and a driving signal were designed during the driving phase. The reset signal was dependent on $V_{Gn}$, f, $t_{R1}$, $t_{R2}$, and $t_{D}$. In the driving stage, the luminance value of EWDs driven by different driving waveforms was used to characterize the performance of driving waveforms.

**Figure 7:**
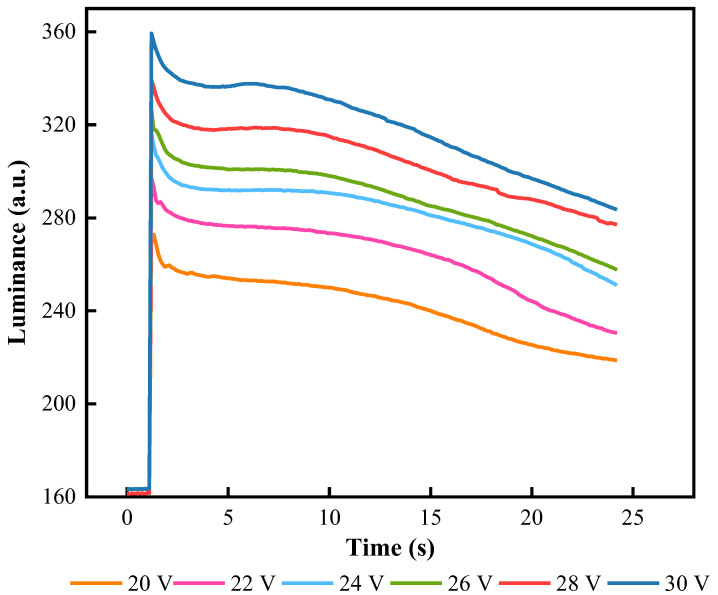
The relationship between luminance and driving time when an EWD was driven by different DC voltages. The curve showed that the drop gradient of luminance was faster with a higher DC voltage. When the DC voltage was 28 V, the EWD was able to maintain a high luminance value.

Variation curves of the luminance driven by different DC voltages are shown in Figure 7. As the DC voltage was increased, the luminance value was also gradually increased. However, when the driving voltage was applied to obtain the maximum luminance value, the display state of the EWD could not be maintained. As the driving time increased, the luminance value gradually decreased. When the DC voltage declined from 30 V to 20 V, the ratio changes of luminance value decreased from 3.28 a.u./s to 2.34 a.u./s within 24 s. Therefore, the driving voltage was positively correlated with the decreasing rate of luminance value. The DC voltage was set to 28 V to obtain a high luminance value.

**Figure 8:**
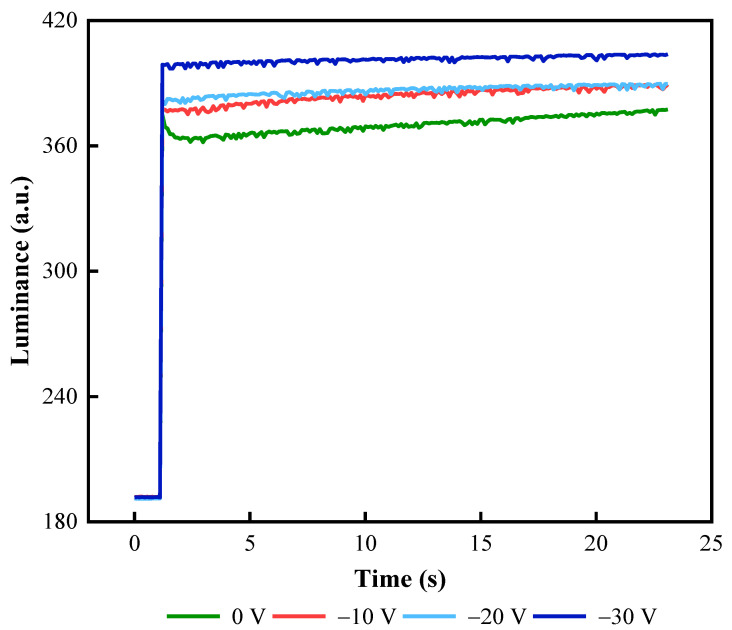
Luminance curves of an EWD driven by reset signals with different negative voltages. There were large fluctuation ranges when the voltage was 0 V, −10 V, and −20 V. Luminance curves were stable when the negative voltage was −30 V.

In the reset signal, the negative voltage during the charge release phase was an important factor in maintaining the stability of the aperture ratio. The frequency of the driving stage was set to 50 Hz. EWD luminance values at different negative voltages are shown in Figure 8. The stability of EWD luminance values was increased significantly with the increase in $V_{Gn}$. Because a high negative voltage could more effectively release trapping charges. Therefore, the negative voltage $V_{Gn}$ was set to −30 V.

**Figure 9:**
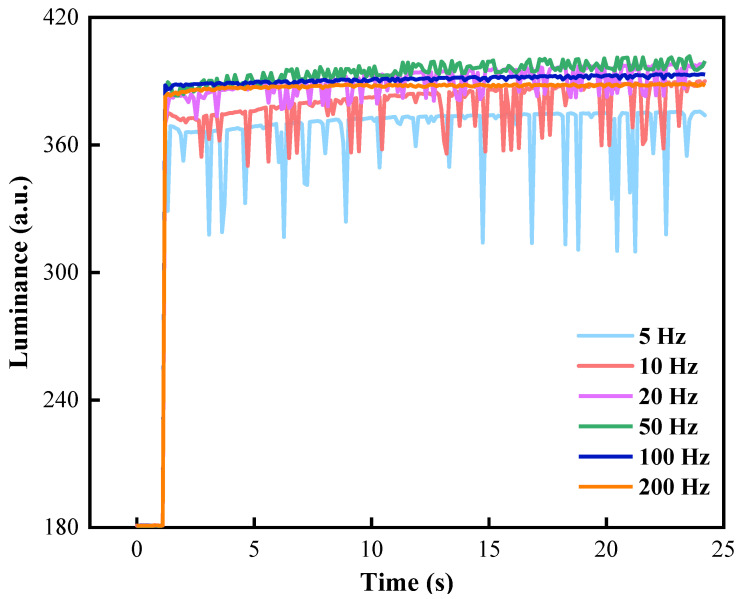
Luminance curves with different frequencies of the driving waveform. There were serious luminance oscillations and a large number of peaks when the frequency was less than 50 Hz. The luminance curve tended to be flat, and the luminance value had a downward trend when the frequency was greater than 100 Hz.

Flickers could be caused by alternating positive and negative voltages at the driving stage, which seriously affected the aperture ratio stability of EWDs. This flaw could be solved by adjusting the frequency of driving waveforms. In the shrinking stage, $t_{1}$ was set to 80 ms and $t_{2}$ was set to 100 ms. In the driving stage, frequencies of driving waveforms were set to 5, 10, 20, 50, 100, and 200 Hz, respectively. Luminance value curves of EWDs at different frequencies are shown in Figure 9. When the driving frequency was less than 20 Hz, the luminance curve had significant oscillations and many peaks, and significant flickers could be observed directly on EWDs. When the driving frequency was increased to 20 Hz and 50 Hz, the number of peaks and the oscillation range were significantly reduced, and significant flickers could not be observed on EWDs. In addition, when driving frequencies were 100 Hz and 200 Hz, the luminance curve tended to be flattened and no peak occurs. However, when the frequency continued to increase, the luminance value showed a downward trend. This was because the negative voltage duration of the charge release stage in the reset signal was insufficient, and charges trapped in the insulating layer could not be fully released. Therefore, the frequency of the driving waveform was set to 100 Hz.

**Figure 10:**
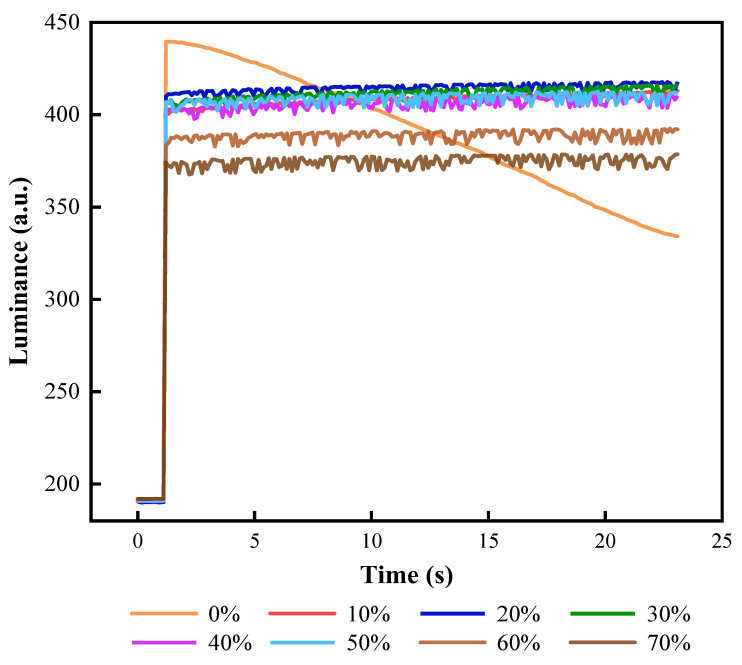
The luminance curve of an EWD driven by reset signals with different η values. The luminance curve significantly decreased when η was 0%. The value of luminance would increase with the increase in η when η was less than 20%. The stability of the luminance curve decreased when η was greater than 30%. The maximum luminance was 420.587 when η was 20%.

A stable aperture ratio of EWDs could be maintained by adjusting the proportion of the reset signal. The luminance curve of different ratios in reset signals is shown in Figure 10. $t_{D}$ was 10 ms because the frequency was set to 100 Hz. Therefore, the proportion of the reset signal could be expressed by Equation (7).

(7) $$\eta =\left(t_{R1}+t_{R2}\right)/t_{D}{}^{\;}$$

When η = 0%, it was equivalent to driving EWDs with a DC voltage, the luminance of the EWD could not be kept stable and decreased significantly at a certain rate. When η was less than 20%, the luminance value was increased without significant jitters. However, when the η was greater than 30%, the luminance value began to decrease gradually, and the oscillation amplitude was increased gradually. The duration of the reset signal was increased, resulting in insufficient driving time for driving a target aperture ratio. To obtain a saturation luminance value and reduce luminance oscillation, η was set to 20%. 

**Figure 11:**
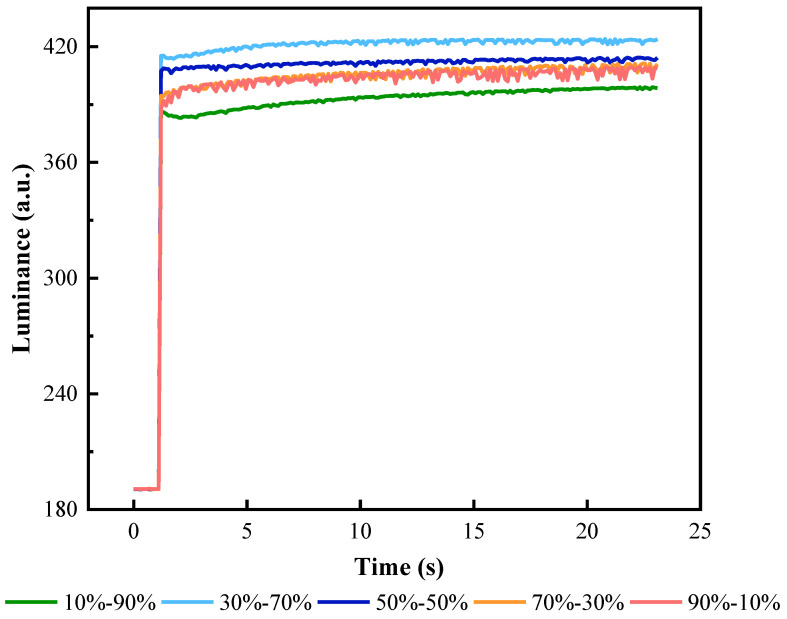
The luminance curve of an EWD with the different ratios of $t_{R1}/t_{R2}$. The oscillation degree of luminance curves was increased with the increase in ρ. The luminance curve had no obvious oscillation and EWDs could keep a stable display when $t_{R1}$ was equal to $t_{R2}$.

In addition, the ratio between $t_{R1}$ and $t_{R2}$ in the reset signal would also affect the stability of oil, which was defined as $\rho =t_{R1}/t_{R2}$. Luminance–time curves at different ρ values are shown in Figure 11. It could be seen that as ρ increased, the oscillation range of luminance and the number of peaks increased. When ρ was less than 1, there was a clear change trend in the luminance curve. It was demonstrated that the luminance value could not be well kept when $t_{R1}$ was less than half of the duration of the reset signal. At the same time, when $t_{R2}$ was less than half the duration of the reset signal, the pixel was difficult to achieve a maximum luminance. Experimental results showed that when $t_{R1}$ was equal to $t_{R2}$, it could obtain a large luminance value and the stability of EWDs was the best.

### 3.5. Performance of the Proposed Waveform

**Figure 12:**
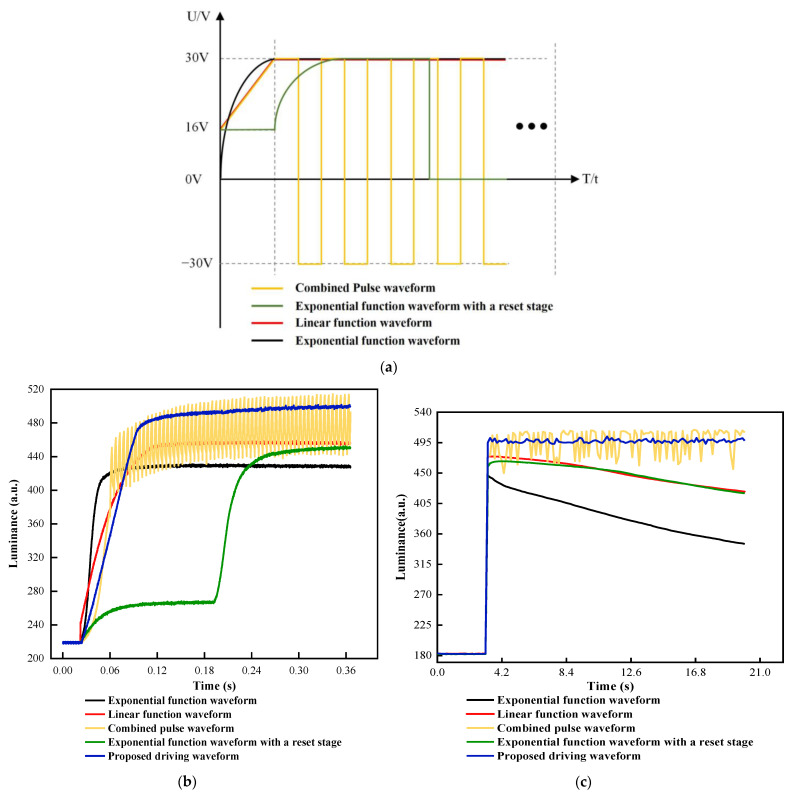
(**a**) Different driving waveforms for performance comparison. The yellow line represents the combined pulse waveform; the green line represents the exponential function waveform with a reset stage; the red line represents the linear function waveform; the blue line represents the traditional exponential function waveform. (**b**) Luminance curves of different driving waveforms in the shrinking stage. The maximum luminance of the proposed driving waveform was 501.567. In addition, the maximum luminance of the combined pulse waveform, the exponential function waveform with a reset stage, the linear function waveform, and the exponential function waveform were 517.137, 452.378, 459.566, and 431.303, respectively. (**c**) Luminance curves of the driving stage. The combined pulse waveform had obvious luminance oscillations, and the oil backflow phenomenon occurred in the other three driving waveforms.

As shown in Figure 12a, four different driving waveforms were used to compare performance with the proposed driving waveform. The four driving waveforms include a combined pulse waveform [20], an exponential function waveform with a reset stage [32], a linear function waveform [19], and an exponential function waveform [33]. In order to control the variable, the duration of the oil shrinking stage in all driving waveforms was set to 180 ms, and the maximum driving voltage $V_{max}$ was set to 30 V. The initial voltage of both the linear function waveform and the exponential function waveform with a reset signal was set to 16 V.

In the shrinking stage, the luminance value of different driving waveforms is shown in Figure 12b. It could be seen that the luminance value of the exponential function waveform increased most quickly, but the saturation luminance value was the lowest compared to the other four driving waveforms. The maximum luminance value was 431.578. It could be proved that an important factor of severe oil splitting was caused by the exponential function waveform. When the luminance was exceeded 450, the luminance of the linear function waveform was increased slowly, and the luminance of the exponential function waveform with a reset signal was gradually stabilized. The maximum luminance value of the linear function waveform and the exponential function waveform were 459.566 and 452.378, respectively. On the contrary, the luminance of the proposed driving waveform and the combined pulse waveform exceeded significantly that of the other three driving waveforms. Their maximum luminance value was 501.567 and 517.137, respectively. The luminance oscillation degree of the proposed driving waveform was lower than that of the combined pulse waveform. This phenomenon showed that the proposed driving waveform not only could effectively reduce the luminance oscillation and the oil splitting but also could increase luminance value and maintain a stable display. Moreover, in the driving stage, the luminance value of different driving waveforms can be seen in Figure 12c. The oil backflow phenomenon occurred in the exponential function waveform, the linear function waveform, and the exponential function waveform with a reset signal because trapped charges were not released. The combined pulse waveform had obvious luminance oscillations, which could not maintain a stable luminance value. The luminance value of the proposed driving waveform did not change significantly, which proved that the charge trapping effect was suppressed. 

Performance parameters of five different driving waveforms and the proposed driving waveform were shown in Table 1. The average luminance of the proposed driving waveform was 497.330, which was higher than the other driving waveforms. The response time of the proposed driving waveform was 103.038 ms, which was 22.74% higher than that of the combined pulse waveform, and 20.27% higher than the linear function waveform. By applying the proposed driving waveform, the aperture ratio could reach 57.43%, which was 7.23% higher than that of the combined pulse waveform. Compared with the exponential function waveform, the aperture ratio was increased by 24.42%. In addition, the luminance standard deviation for the proposed waveform was 2.3428, which was lower than the other four driving waveforms. Compared with the exponential function waveform with a reset signal, the luminance standard deviation was increased by 84.61%. The experiment results showed that the charge trapping effect was effectively suppressed by applying the proposed driving waveform. Therefore, the proposed driving waveform in this study could solve the oil backflow of EWDs and keep a stable oil display state.

## 4. Conclusions

In order to suppress the charges trapping effect, a new combined driving waveform was proposed in this paper. Firstly, a combined waveform based on an exponential function and a DC voltage was designed in the oil shrinkage stage by analyzing the principle of EWDs and measuring voltage characteristic curves of EWDs. Then, oil splitting was suppressed by quickly breaking and fully combining oil. At the same time, an optimized AC reset waveform was designed in the oil driving stage to suppress the oil backflow and increase the average luminance of EWDs. Compared with other driving waveforms, oil backflow could be effectively suppressed, and luminance oscillation of EWDs was decreased, a more stable aperture ratio could be maintained by the proposed driving waveform. In summary, the proposed driving waveform provided a certain reference value for improving the performance of EWDs. 

## Figures and Tables

**Table 1:** Driving effect comparison when different driving waveforms were used to drive EWDs.

Driving Waveform	Combined [20]	Reset Exponential Function [30]	Linear Function [31]	Exponential Function [32]	Proposed
Average (a.u.)	495.766	448.530	450.308	387.648	497.330
Standard deviation	20.6558	15.2184	16.9525	28.3286	2.3428
Response time (ms)	133.358	260.1	129.228	73.205	103.038
aperture ratio (%)	53.56	49.52	50.84	46.16	57.43

## Data Availability

Data are contained within the article.
